# Electrolytic Cleaning of Dental Implants: A Scoping Review of Clinical Studies

**DOI:** 10.3390/dj13040172

**Published:** 2025-04-18

**Authors:** Anastasia Klein, Giulio Rasperini, Reinhard Gruber, Oleh Andrukhov, Xiaohui Rausch-Fan

**Affiliations:** 1 Clinical Division of Periodontology, University Clinic of Dentistry, Medical University of Vienna, 1090 Vienna, Austria; anastasia.klein@meduniwien.ac.at; 2 Department of Biomedical, Surgical and Dental Sciences, Università Degli Studi di Milano, 20100 Milano, Italy; giulio.rasperini@unimi.it; 3 Competence Center for Oral Biology, University Clinic of Dentistry, Medical University of Vienna, 1090 Vienna, Austria; reinhard.gruber@meduniwien.ac.at; 4 Competence Center for Periodontal Research, University Clinic of Dentistry, Medical University of Vienna, 1090 Vienna, Austria; oleh.andrukhov@meduniwien.ac.at; 5 Center for Clinical Research, University Clinic of Dentistry, Medical University of Vienna, 1090 Vienna, Austria

**Keywords:** electrolytic cleaning, electrolytic decontamination, dental implants, peri-implantitis, peri-implant disease, GalvoSurge

## Abstract

**Background/Objectives:** This literature review aims to systematically analyze the efficacy of electrolytic cleaning for treating peri-implantitis, including its impact on disease resolution, re-osseointegration of treated implants, and peri-implantitis recurrence. It also compares various study and treatment protocols used in the selected papers. **Methods:** A comprehensive search was conducted in MEDLINE (via PubMed) and the Cochrane Central Register of Controlled Trials using the keywords “electrolytic cleaning implant” or “GalvoSurge”. Studies published until 31 December 2024 were considered for inclusion. **Results:** Out of 141 articles retrieved, four publications were selected for the review. These studies were analyzed for implant type, number, evaluation methods, observation periods, surgical procedures, and additional treatments. Disease resolution was reported in one study, while peri-implantitis recurred in the remaining studies. However, re-osseointegration of treated implants was observed in all selected papers. **Conclusions:** Due to the limited and heterogeneous nature of the studies, it is difficult to draw definitive conclusions about the effectiveness of electrolytic cleaning as a treatment for peri-implantitis. To ensure consistent trial outcomes and improve predictability, clear clinical guidelines and surgical protocols for electrolytic decontamination are essential.

## 1. Introduction

Dental implants have been used to replace missing teeth for many decades [1]. Until now, titanium implants have been considered the gold standard, primarily due to their exceptional biocompatibility and the ability to achieve osseointegration [2]. The following characteristics appear to play a crucial role in ensuring long-lasting and direct bone-to-implant contact: implant material, implant design, bone quality, implant loading, and finishing.

The role of implant surface characteristics rose to prominence in the early 1980s, when Albrektsson et al. suggested that the biological response of the body correlated with the implant surface type [2], which seems to affect primary interfacial reactions with blood components, bone, epithelial, and connective tissue cells as part of a wound healing process [3,4,5].

Two factors have been identified to play a possible role in the histological and histometric differences between hydrophilic and hydrophobic implant surfaces during early reactions of wound healing: The first factor relates to different patterns of adsorbed plasma proteins that cause specific up- or down-regulations of gene expression of the adjacent progenitor cells [6,7,8]. The second describes a more stabilized blood clot on hydrophilic surfaces [9]. There is also enhanced angiogenesis on hydrophilic surfaces during the early stages of osseointegration [9], which is known to be beneficial for bone formation. The current standard considers these interrelating factors [10] and several approaches; that is, GalvoSurge^®^ [11,12] can be implemented to turn hydrophobic implant surfaces into hydrophilic ones.

Nowadays, long-term survival rates of dental implants are considered excellent. Nevertheless, in approximately 5% of patients, secondary implant failure is diagnosed years after a successful osseointegration. It is commonly caused by peri-implantitis [13,14], which has become a significant and rapidly growing problem in dentistry [15,16]. According to a recent systematic review and meta-analysis, the prevalence of peri-implantitis was estimated at 19.53% at the patient level and 12.53% at the implant level [17].

Multiple non-surgical decontamination approaches have been proposed and tested to treat peri-implantitis [18], but these demonstrate only limited efficacy in obtaining disease resolution when using non-surgical mechanical peri-implantitis treatment alone or as a combination with adjunctive or alternative measures [19,20].

There are two main approaches to treating peri-implantitis surgically: non-augmentative and augmentative procedures [21]. The success of these methods varies considerably in terms of arresting further progression of peri-implant disease [22,23,24,25,26,27,28]. Thus, none of the existing surgical protocols can be considered the best treatment modality for peri-implantitis [21].

Electrolytic cleaning is a novel approach to decontaminate dental implants without changing their surface microtopography and affecting their physical properties [28]. GalvoSurge^®^ is currently the only device available on the market that employs this method to clean dental implants, utilizing sodium formate as an electrolytic solution. During the cleaning process, titanium implants are loaded with a maximum current of 600 mA, and an electrolyte solution is pumped through a platinized ring. As a result, carbon atoms are removed, which improves the surface bioactivity of the implants by converting them back into a hydrophilic one. This should lead not only to better attachment of bone cells but also to the re-osseointegration of the implants [11,12]. Thus, electrolytic cleaning represents a promising approach for treating peri-implantitis. The present scoping review aims to map the evidence on the clinical effectiveness of this approach and the challenges associated with its clinical application.

## 2. Materials and Methods

### 2.1. Method

An electronic literature search was conducted in two databases according to the PRISMA-ScR guidelines: the National Library of Medicine (MEDLINE via PubMed) and the Cochrane Central Register of Controlled Trials. The following search strategy was used: “electrolytic cleaning implant OR GalvoSurge”. The literature search was conducted up to and including 31 December 2024. The research question was formulated as follows: How does electrolytic cleaning (I—Intervention) in patients with peri-implantitis (P—Patients) improve clinical outcome (O—outcome)?

### 2.2. Selection of Papers

The following inclusion criteria were applied: clinical studies, clinical trials, or case reports, patients with peri-implantitis and titanium dental implants, in which electrolytic decontamination using an electrolytic solution is applied as an adjuvant therapy, English language, and full-text availability. The selection of the relevant publications consisted of two phases. In the first phase, all abstracts that appeared in the search results were analyzed to determine whether they met the primary inclusion criteria. In the second phase, all the available full texts of the shortlisted abstracts were evaluated, and irrelevant studies were eliminated. The information collected from all the remaining shortlisted papers was then systematized in a tabular form. Manuscript selection and data extraction were performed independently by two researchers (A.K. and O.A.) using a standardized data extraction form. The following parameters were extracted: study design and characteristics, details on the patients’ population, description of the intervention, outcome, and main findings. Discrepancies between the researchers were resolved by consulting with a third researcher (X.R.-F.).

### 2.3. Tool for Risk of Bias Assessment

Risk of bias assessment was performed using the RoB 2 tool [29].

## 3. Results

### 3.1. Article Selection

**Figure 1:**
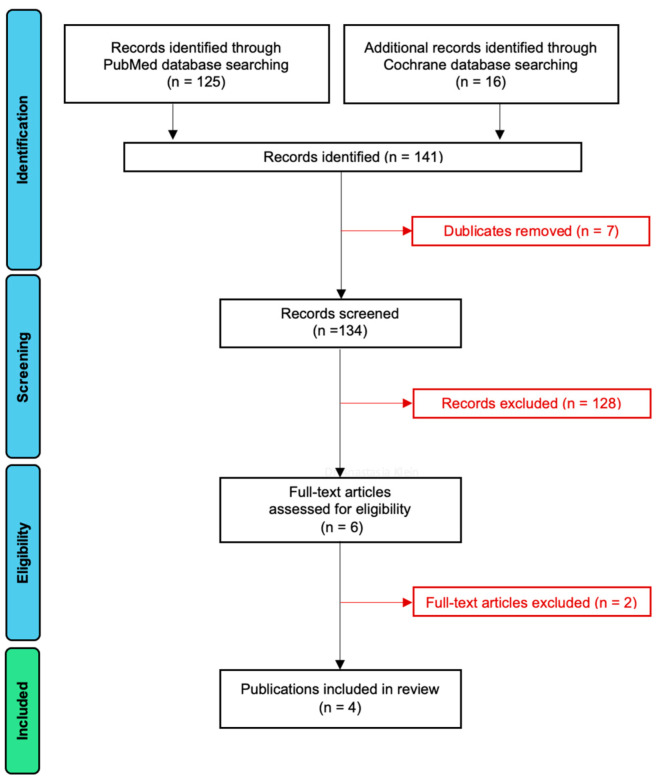
PRISMA flowchart.

The PRISMA flowchart of the article selection process is presented in Figure 1. A total of 125 papers were found in the PubMed database, and 16 studies were identified in the Cochrane Central Register of Controlled Trials. After seven duplicates had been removed, the titles and abstracts of 134 records were screened. As a result of the screening, only 6 papers remained, and 128 records had to be eliminated as they did not fulfill the inclusion criteria. After assessing the eligibility of these six publications, two were excluded because they did not use GalvoSurge. The four remaining articles were included in this systematic review [30,31,32,33]. The data from these papers are extracted and systematized in Table 1, Table 2, Table 3, Table 4 and Table 5.

### 3.2. Study Design, Number of Patients and Implants, and Evaluation Period

The basic characteristics of the studies included in the review are summarized in Table 1. Two of the papers were case reports [30,31] and the other two were randomized controlled clinical trials [31,32]. The primary objective of the shortlisted studies was to evaluate clinical outcomes and re-osseointegration after electrolytic cleaning and regenerative therapy of dental implants with peri-implantitis. All of the clinical trials utilized GalvoSurge^®^ to clean the implants by means of electrolysis.

The case report of Gianfreda et al. had only one patient with one implant [30]. The second case report by Bosshardt et al. [31] reported the application of electrolytic cleaning procedures on three patients who had developed peri-implantitis around four implants [31]. In this study, the implants were removed due to recurrent peri-implantitis and analyzed histologically. Two other papers were randomized clinical trials and included 24 patients with 24 implants in total [31,32].

In all selected studies, various implant types and designs were observed (Table 1), ranging from blasted, anodized, and etched implants to implants with an HA coating. Bone-level and tissue-level implants were treated in the same manner.

The case report study by Gianfreda et al. had the longest observation period of two years [30]. The shortest period of 6 months was described in the randomized controlled clinical trial by Schlee et al. [32], whereas Bosshardt et al. had an incoherent evaluation period ranging from 6 to 13 months [31].

### 3.3. Patients’ Characteristics and Treatment Protocol

Patients’ characteristics and peri-implantitis treatment protocols are summarized in Table 2.

#### 3.3.1. Periodontal and Peri-Implant Disease

All studies stated that all treated implants had peri-implantitis [30,31,32,33]. In the case report by Gianfreda et al., the patient had undergone a 6-month maintenance therapy prior to surgery, but the history of periodontitis was not reported [30]. Bosshardt et al. provided no information on whether patients suffered from periodontal disease before the electrolytic treatment [31]. Both randomized clinical trials had no patients with uncontrolled periodontitis [32,33].

#### 3.3.2. Initial Clinical Findings

The following initial clinical findings were documented in three out of four publications [30,32,33]: bleeding on probing (BoP), plaque index (PI), implant pocket depth (PD), gender, age, and smoking habits. Two papers reported suppuration before the treatment [32,33]. The case report by Gianfreda et al. presented a periodontal chart with exact measurements and the location of bleeding points, as well as plaque accumulation sites in the whole mouth of the patient [30]. This was also the only study where CBCT was carried out, and bone deficit around the affected implant was measured digitally prior to surgical and electrolytic treatments. The case report by Bosshardt et al. provided information on implant pocket depth only [31]. The randomized controlled clinical trials stated that bleeding on probing and plaque indices were less than 20%, without specifying the exact location [32,33].

#### 3.3.3. Treatment Protocol

##### Suprastructure Removal

In all studies, suprastructures were removed prior to surgery [30,31,32,33]. Gianfreda et al. removed the implant crown 1 week after the professional hygiene session, which involved decontamination of the implant area with AirFlow Master Piezon^®^ and application of Periostat^®^ gel [30]. After that, a cover screw was inserted, iodoform paste was applied, and a Maryland bridge was placed. Schlee et al. removed superstructures, cleaned the affected area with PerioFlow^®^ (erythritol), rinsed it with chlorhexidine, and placed cover screws [32,33]. Bosshardt et al. removed the suprastructures immediately before the surgery [31].

##### Timing of Surgery and Electrolytic Cleaning

Surgery and electrolytic cleaning were performed either immediately after prosthesis removal [31] or 2 [32,33] to 4 weeks later [30]. Bosshardt et al. performed electrolytic cleaning and bone augmentation procedures on the day of the suprastructure removal [31]. Schlee et al. cleaned the implants with a GalvoSurge^®^ device and augmented the area using GBR 14 days after the suprastructure removal [32,33]. Gianfreda et al. had the longest period between removing the implant crown and performing the regenerative surgery—4 weeks [30].

##### Evaluation Methods

Probing depth (PD), bleeding on probing (BoP), and radiographs were used in all studies [30,31,32,33]. The distance from the implant platform to the most apical position of the bone (P-B) was measured at 6 points (m, mb, b, db, d, dl) in three studies [31,32,33]. Bone-level implants were considered completely osseointegrated if the bone level reached the platform. Implants with polished necks were counted as complete bone fill if the bone fill reached the border rough-polished [32,33].

Removed implants were analyzed histologically and histomorphometrically only by Bosshard et al. [31]. Here, the base of the former bone defect was determined by the demarcation line interfacing with old bone in the apical part of the implant and newer bone in the implant’s more coronal part. Gianfreda et al. applied CBCT to evaluate bony defects around the treated implant [30]. Statistical analysis was performed only by Schlee et al. [32,33].

### 3.4. Surgical Protocols

The details of surgical protocols in all studies are presented in Table 3.

#### 3.4.1. Flap Design

Gianfreda et al. made a crestal incision with special additional incisions to create a surgical papilla [30]. A split-thickness flap was raised, followed by a full-thickness flap. Then, lateral extension incisions were performed, and anatomic papillae were de-epithelialized. Schlee et al. mentioned crestal incisions with a vertical extension of the flap [32,33]. Additionally, buccal periosteal incisions in the upper jaw and both buccal and lingual periosteal incisions in the lower jaw were made without specifying the thickness and/or borders of the flap. Bosshardt et al. provided no information on their flap design [31].

#### 3.4.2. Bone Augmentation

All studies reported the use of a combination of autogenous bone and bone substitutes [30,31,32,33]. Three implants treated by Bosshardt et al. received a 50:50 mix of autogenous bone and BioOss^®^; a bony defect around the fourth implant was augmented with a 50:50 mix of autogenous bone and Maxigraft^®^ [31]. Schlee et al. had the same percentage of autogenous bone substitutes; the substitute of their choice was BioOss^®^ [32,33]. The same bone substitute brand was used by Gianfreda et al., without specifying the amount of xenogenic material that was mixed with the autogenous bone of the patient [30].

#### 3.4.3. Barrier Membrane

Barrier membranes were placed in all identified studies to facilitate GBR. Schlee et al. [32,33] as well as Bosshardt et al. [31] chose BioGide^®^ collagen membranes to cover the augmented areas, and only one implant received Jason^®^ collagen membrane instead [31]. Gianfreda et al. mentioned performing the GTR procedures with the help of Cytoplast Ti-250^®^ membrane [30].

#### 3.4.4. Membrane Fixation Method

Gianfreda et al. stabilized the membrane with two mini-screws [30]. Neither Bosshardt et al. nor Schlee et al. provided exact information on how many screws were used per implant, only the approximate number of screws and the screw brand (Umbrella screws, Ustomed, Tuttlingen, Germany) were reported [31,32,33].

#### 3.4.5. Suture

Schlee et al. used Propylene monofilament 6.0 (Medipac^®^, Kilis, Greece) sutures [32,33]; Gianfreda et al. had PTFE 5.0 (Omnia^®^, Fidenza, Italy) for mattress sutures on the implant area and PGCL 6.0 (Monofast^®^, Kirkinil, Greece) for detached and sling stitches on the papillae [30]. There is no information on suturing material in the study by Bosshardt et al. [31].

### 3.5. Mechanical Decontamination, Antiseptic and/or Antibiotic Treatment

The details on implant decontamination and antibacterial treatments in all studies are presented in Table 4.

#### 3.5.1. Powder Spray System

Gianfreda et al. used AirFlow Master Piezon^®^ 1 week prior to implant crown removal [30]. Schlee et al. utilized PerioFlow^®^ erythritol after suprastructure removal 14 days before the surgery [32,33]. Additionally, AirFlow Plus^®^ was applied in the control group in this trial. No powder spray system was mentioned in the case report study conducted by Bosshardt et al. [31].

#### 3.5.2. Antiseptics

Three out of four papers used chlorhexidine before and/or after the surgery [30,32,33]. Gianfreda et al. applied iodoform paste before cover screw placement and prescribed Curasept^®^ 0.12% twice daily for 2 weeks after the surgery [30]. The patients in the randomized controlled clinical study received Chlorhexamed^®^ 0.2% 2 weeks before surgery, and in case of implant exposure, after surgery [32,33]. One study did not provide any information on antiseptics [31].

#### 3.5.3. Antibiotics

Only one trial reported the use of antibiotics: Periostat^®^ gel was applied via a carrier with a tip within the defect after professional oral hygiene treatment and decontamination of the implant area with erythritol aerosol (Airflow Master Piezon^®^, EMS, Nyon, Switzerland) [30]. Before the electrolytic cleaning with GalvoSurge^®^, the defect was degranulated and washed with rifampicin (Rifadin^®^, Sanofi, Milan, Italy). Additionally, 875 mg of amoxicillin and 125 mg of clavulanic acid were prescribed for 4 days, twice daily, after the surgery.

### 3.6. Study Results

The outcome of all studies included in this review is summarized in Table 5.

#### 3.6.1. Disease Resolution

Only one study diagnosed disease resolution after electrolytic cleaning [28]. According to the authors of this study, no clinical problems were detected, and periodontal indices remained stable.

#### 3.6.2. Re-Osseointegration

All studies stated that re-osseointegration of the treated implants was observed [30,31,32,33], but only one of these trials could confirm it histologically and histomorphometrically after the implants had been removed due to recurrent peri-implantitis [31].

#### 3.6.3. Peri-Implantitis Recurrence

Peri-implantitis reoccurred in three trials after electrolytic decontamination [31,32,33]. In one of these studies, recurrent peri-implantitis was diagnosed during the follow-ups, and all implants had to be removed with a trephine bur [31]. Only 18 out of 24 treated implants could be assessed in a randomized clinical trial by Schlee et al., as 6 implants were re-infected and needed to be extracted [32,33].

### 3.7. Risk of Bias Assessment

**Figure 2:**
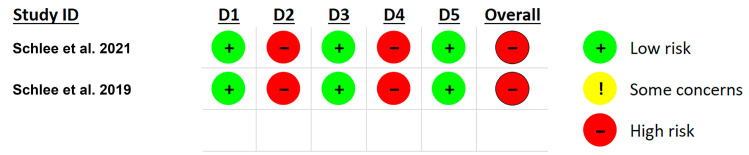
Risk of bias was assessed using the RoB2 tool in the following domains: D1—randomization process; D2—deviations from the intended interventions; D3—missing outcome data; D4—measurements of the outcome; D5—selection of the reported results [32,33].

Risk of bias assessment was made only for the two clinical trials involved in this scoping review, and these results are presented in Figure 2. The significant bias in both clinical trials was associated with the fact that both patients and clinicians were aware of the treatment. Therefore, both studies gave a high overall risk of bias.

## 4. Discussion

The clinical trials reviewed in this paper focused exclusively on peri-implantitis sites. The primary objective of peri-implantitis treatment is to resolve the disease. While all authors of the four analyzed clinical studies [30,31,32,33] referenced the 2017 World Workshop on the Classification of Periodontal and Peri-Implant Diseases and Conditions and Berglundh et al. for diagnosing peri-implantitis [34], none specified the exact criteria used to define peri-implantitis resolution.

In their study, Monje et al. proposed two definitions for the resolution of peri-implantitis: “dogmatic” and “flexible” [35]. The “dogmatic” definition requires the following criteria: no bleeding and/or suppuration on gentle probing (~0.2 N), probing pocket depths of ≤5 mm, and no radiographic evidence of progressive bone loss greater than the standard error of ≥1 mm [36]. Alternatively, the “flexible” definition includes ≤2 dots of light bleeding on gentle probing (~0.2 N), no suppuration on gentle probing (~0.2 N), probing pocket depths of ≤5 mm, and no radiographic evidence of progressive bone loss beyond the standard error of ≥1 mm [36]. These criteria could be valuable for future clinical trials assessing the success of peri-implantitis treatments.

Studies have shown that measurements obtained with CBCT demonstrate a strong correlation with histomorphometric data of the vestibular bone, oral bone thickness, and implant length. Furthermore, it provides precise information about the bone in all dimensions around the implant, allowing for an accurate analysis of the bone structure directly at the implant surface [37,38]. Gianfreda et al. was the sole study among the clinical trials reviewed in this paper that employed CBCT to evaluate bone levels around the affected implant [30]. In future research, CBCT may prove to be a more dependable diagnostic tool for assessing bone levels in peri-implantitis cases.

Schwarz et al. (2007) and Monje et al. (2019) introduced two classification systems for various bony defects around dental implants affected by peri-implantitis [39,40]. Additionally, Schlee et al. developed their own classification based on the regenerative potential of these defects [33]. Notably, this system was the only one used in their randomized controlled trial (RCT) analyzed in this paper.

GBR was performed in all the clinical studies reviewed after electrolytic cleaning to address bony defects, regardless of the defect type. This approach made it difficult to compare and analyze the study results statistically [30,31,32,33]. Therefore, a standardized peri-implantitis defect morphology classification system would be valuable not only for future scientific research but also for clinicians when planning surgical procedures to treat patients with peri-implantitis.

A combination of electrolytic decontamination with implantoplasty might be a necessary procedure, but neither official recommendations nor clinical studies exist in this regard. To our knowledge, no clinical studies on electrolytic cleaning of dental implants without augmenting the bony defects afterward have been published so far. Therefore, no data on the effects of electrolytic decontamination on surrounding tissues exists. Consequently, it is not possible to determine the influence of this procedure on different bone substitutes, and more research needs to be conducted.

Re-osseointegration can only be confirmed through histomorphometry, a reliable diagnostic method for assessing the bone–implant interface, material safety, biocompatibility, and tissue reactions. This technique enables detailed qualitative and quantitative analysis of undecalcified bone samples [41]. As a result, it is challenging to determine the extent of osseointegration of the augmented bone clinically. Notably, only one of the reviewed studies conducted histomorphometric analysis on explanted implants that were removed due to recurrent peri-implantitis [31].

The high rate of reported exposures [31,32,33] suggests that the flap designs and/or materials used in the studies might not have been ideal for this type of intervention. A different flap design and a more rigid membrane and/or bone graft substitute type might be more beneficial for these kinds of clinical cases. There are no studies on hand currently that would compare different flap designs, bone substitute types, and/or membranes for peri-implantitis treatment in terms of wound stability and implant exposure rates.

To evaluate the decontamination properties of the GalvoSurge^®^ device, all the studies [31,32,33] followed a protocol where suprastructures were removed, and cover screws were placed prior to the planned surgery. After treatment, the sites were left to heal submerged to prevent re-infection, although there is no scientific evidence supporting the benefits of this approach. Many current peri-implantitis cases involve cemented prosthetic restorations that cannot be removed without causing damage. Future improvements in the design of the GalvoSurge^®^ spray head and/or the protocol for electrolytic cleaning of infected implants could address these challenges.

Summarizing the studies included in this scoping review, we can conclude that a universally standardized classification system for various bony defects around dental implants affected by peri-implantitis would enhance patient case selection for electrolytic cleaning, enabling better comparison and analysis of cases and making the testing of electrolytic decontamination more efficient. Furthermore, incorporating CBCT in all peri-implantitis clinical trials to assess bone levels around the affected implant before and after treatment would provide a more accurate statistical analysis of treatment outcomes. This, in turn, would facilitate the development of more effective treatment protocols.

Our scoping review has several limitations. First, we focused solely on the approach using an electrolytic solution, while other devices (e.g., X-Implant^®^) that also use electrolytic cleaning principles were not considered. Second, we searched the literature in only two databases—PubMed and the Cochrane Central Register of Controlled Trials—and did not include gray literature.

## 5. Conclusions

While electrolytic cleaning of dental implants has shown promising potential, establishing clear clinical guidelines and standardized surgical protocols is crucial for achieving predictable outcomes. However, this can only be accomplished through additional well-designed clinical studies. Our review aims to contribute to the quality assessment of existing research on electrolytic decontamination. The findings presented here can help optimize current surgical protocols and guide the planning of future trials.

## Figures and Tables

**Table 1:** Basic characteristics of studies included in the review.

Study	Study Design	No. Patients	No. Implants	Implant Type	Evaluation Period
Gianfreda et al., 2022 [30]	Case Report	1	1	1 Bredent, Senden, Germany	2 years
Bosshardt et al., 2022 [31]	Case Report	3	4	1 Steri Oss HA-coated 3,8/12 mm 1 Straumann BL, RC, SLActive 4,1/12 mm 1 Straumann BL, RC, SLActive, Ti + G3 4,8/12 mm 1 Straumann BL, RC, SLActive, Ti 4,1/14 mm	6–13 months
w	Randomized Controlled Clinical Trial	24	24	5 Astra TX 2 Astra EV 2 Straumann tissue level 1 Straumann bone level 2 Conelog 2 Camlog 2 Ankylos 1 Sky 2 Branemark 1 Xive 1 Steri Oss 2 Zimmer 1 Nobel Active	18 Months
Schlee et al., 2019. [32]	Randomized Controlled Clinical Trial, Proof of Principle	24	24	5 Astra TX 2 Astra EV 2 Straumann tissue level 1 Straumann bone level 2 Conelog 2 Camlog 2 Ankylos 1 Sky 2 Branemark 1 Xive 1 Steri Oss 2 Zimmer 1 Nobel Active	6 Months

**Table 2:** Clinical protocols used in the studies included in the review.

Study	Diagnosis	Initial Clinical Findings	Treatment Protocol	Evaluation Methods
Gianfreda et al., 2022 [30]	Peri-implantitis	Good periodontal health; 6-month maintenance; Periodontal chart 62 y.o.; Male; BoP 0%; PI 5%; Implant PD 9 mm; Implant CBCT 5.5 mm Bone deficit;	Periapical X-ray, PD, CBCT T00: Professional oral hygiene + Periostat^®^ gel T0 (1 week): Implant crown removal; Cover screw insertion + iodoform paste; Maryland bridge T1 (4 weeks): Defect degranulation + rifampicin; Electrolytic cleaning + GBR + platelet aggregates	Periapical X-ray Probing depth (PD) CBCT Bleeding on probing (BoP)
Bosshardt et al., 2022 [31]	Peri-implantitis	55–62 y.o.; Females Implant PD	T0: Suprastructure removal; PD, BoP, radiographs; Electrolytic cleaning + GBR; Cover screw placement T1 (6 months): Suprastructure placement; PD; BoP; radiographs; T2 (13 months): PD; BoP; radiographs; explantation	Probing depth (PD): 6 points (m, mb, b, db, d, dl) Bleeding on probing (BoP): 6 points (m, mb, b, db, d, dl) P-B distance: 6 points (m, mb, b, db, d, dl) Radiographic bone level Histology Histomorphometry
Schlee et al., 2021 [33]	Peri-implantitis	No periodontitis Mean age 50% male/50% female BoP < 20% PI < 20% Implant BoP 100% Implant Pus 100% Implant mean PD < 10 cigarettes/day	Test group (12 patients; 12 implants): electrolytic cleaning Control group (12 patients; 12 implants): powder spray + electrolytic cleaning T2 (after 12 months): PD; BoP; pus; recessions; photos; radiographs T3 (after 18 months): PD; BoP; pus; recessions; photos; radiographs	Probing depth (PD): 6 points (m, mb, b, db, d, dl) Bleeding on probing (BoP): 6 points (m, mb, b, db, d, dl) P-B distance: 6 points (m, mb, b, db, d, dl) Radiographic bone level Statistical analysis
Schlee et al., 2019 [32]	Peri-implantitis	No periodontitis Mean age 50% male/50% female BoP < 20% PI < 20% Implant BoP 100% Implant Pus 100% Implant mean PD < 10 cigarettes/day	Test group (12 patients; 12 implants): electrolytic cleaning Control group (12 patients; 12 implants): powder spray + electrolytic cleaning T00: Suprastructure removal; PSS cleaning + chlorhexidine; Cover screw placement T0 (14 days after): Photos; PPD; BoP; pus; radiographs; Electrolytic cleaning & GBR T1 (6 months after): Photos; radiographs; P-D distance; BoP; pus, recessions; Exposed parts cleaned with cleaning paste; Suprastructure placement	Standardized photos (occlusal, buccal, lingual view) Probing depth (PD): 6 points (m, mb, b, db, d, dl) Bleeding on probing (BoP): 6 points (m, mb, b, db, d, dl) P-B distance: 6 points (m, mb, b, db, d, dl) Bone gain VAS assessment

**Table 3:** Surgical protocols used in the studies included in the review.

Study	Flap Design	Bone Augmentation	Membranes	Pins/Miniscrews	Suture
Gianfreda et al., 2022 [30]	Crestal incision Incisions to draw surgical papillae Flap dissection at half thickness up to the amelocemental junction and a full thickness apically to it De-epithelialization of the anatomic papillae Lateral extension of the incisions	Autogenous bone and BioOss^®^	Cytoplast Ti-250^®^	2 mini-screws	PTFE 5.0 PGCL 6.0
Bosshardt et al., 2022 [31]	No information	50:50 autogenous bone and 3 BioOss^®^ 1 Maxgraft^®^	3 BioGide^®^ 1 Jason^®^	0/2/3 umbrella screws	No information
Schlee et al., 2021 [33]	Crestal incision with releasing vertical incisions Maxilla: buccal periosteal incisions Mandible: buccal and lingual periosteal incisions	50:50 autogenous bone and BioOss^®^	BioGide^®^	Umbrella screws	Propylene monofilamens 6.0
Schlee et al., 2019 [32]	Crestal incision with releasing vertical incisions Maxilla: buccal periosteal incisions Mandible: buccal and lingual periosteal incisions	50:50 autogenous bone and BioOss^®^	BioGide^®^	Umbrella screws	Propylene monofilamens 6.0

**Table 4:** Implant decontamination protocol and antibacterial treatment in the studies included in the review.

Study	Powder Spray System	Antiseptics	Antibiotics
Gianfreda et al., 2022 [30]	AirFlow Master Piezon^®^ (1 week before implant crown removal)	Iodoform paste (before cover screw placement) Curasept^®^ 0.12% (twice daily for 2 weeks after surgery)	2 mini-screws
Bosshardt et al., 2022 [31]	No	No	No
Schlee et al., 2021 [33]	PerioFlow^®^, erythritol, EMS^®^ (after suprastructure removal, 2 weeks before surgery) AirFlow Plus^®^, EMS^®^ for control group	Chlorhexamed Forte^®^ 0.2% (2 weeks before surgery)	No
Schlee et al., 2019 [32]	PerioFlow^®^, erythritol, EMS^®^ (after suprastructure removal, 2 weeks before surgery) AirFlow Plus^®^, EMS^®^ for control group	Chlorhexamed Forte^®^ 0.2% (2 weeks before surgery and in case of exposure after surgery)	No

**Table 5:** Outcome of the studies included in the review.

Study	Results	Disease Resolution	Re-Osseointegration	Peri-Implantitis Recurrence
Gianfreda et al., 2022 [30]	No clinical problems Stable periodontal indices Bone gain	Yes	Yes	No
Bosshardt et al., 2022 [31]	Radiographic and histological bone gain; Reduced PD and BoP	No	Yes	Yes
Schlee et al., 2021 [33]	18 implants assessed Statistically significant radiologic bone gain compared to T0; T2 and T3: No statistically significant change in BoP and Pus; Significant PD reduction; Cleaning with PSS: no additional benefit	No	Yes	Yes
Schlee et al., 2019 [32]	Bone gain: no statistically significant difference between groups Significant clinical bone fill	No	Yes	Yes

## Data Availability

Data sharing is not applicable.

## Abbreviations

The following abbreviations are used in this manuscript:

BL	Bone level
BoP	Bleeding on probing
CBCT	Cone beam computed tomography
GBR	Guided bone regeneration
GTR	Guided tissue regeneration
HA	Hydroxyapatite
PI	Plaque index
PD	Probing depth
P-B	Distance from the implant platform to the most apical position of bone
PGCL	Polyglycolic acid copolymer
PRISMA	Preferred Reporting Items for Systematic Reviews and Meta-Analyses
PSS	Powder spray system
PTFE	Polytetrafluoroethylene
RC	Regular connection
RCT	Randomized controlled trial
SLA	Sandblasted, large-grid, acid-etched
Ti	Titanium
T00	Pre-treatment baseline
T0	Initial time point
T1	First time point
T2	Second time point
T3	Third time point
